# ID 300 - Produtividade Perdida Associada a Doenças Negligenciadas na América do Sul (2019)

**DOI:** 10.5327/2237-9622.2025.v34s1.300

**Published:** 2025-11-25

**Authors:** Thaís Pereira Catão, Cid Manso de Mello Vianna, Gabriela Bittencourt Gonzalez Mosegui

**Keywords:** doenças tropicais negligenciadas, perda de produtividade permanente, efeitos psicossociais da doença

## Abstract

**Introdução:**

As doenças tropicais negligenciadas (DTNs) são um grupo de doenças infecciosas que predominam em áreas tropicais e subtropicais e que afetam desproporcionalmente os indivíduos que vivem em condições de pobreza. Tais doenças causam consequências sanitárias, sociais e econômicas devastadoras para mais de um bilhão de pessoas e estão, muitas vezes, associadas às condições ambientais. Este estudo estimou a perda de produtividade relacionada à mortalidade prematura em 2019 por DTNs para os países da América do Sul (AS). A estimativa da perda de produtividade ajuda a dimensionar o dano causado pela falta de investimento das agências financiadoras de Ciência e Tecnologia (C&T) e da indústria farmacêutica em seus tratamentos. Também permite mensurar o impacto monetário e social das condições precárias de saúde, frequentemente ligadas à pobreza e à falta de acesso a serviços de saúde.

**Método:**

Utilizaram-se dados de mortalidade para estimar a produtividade perdida permanente, usando uma da abordagem do capital humano (ACH). Os dados de mortalidade foram retirados do (GBD) 2019, no site eletrônico do (IHME). Foram realizadas análises de correlação entre variáveis socioeconômicas, demográficas e de saúde dos países, extraídas de bases de dados como: Associação Internacional de Seguridade Social (ISSA), Comissão Econômica para a América Latina e Caribe (Cepal), e Banco Interamericano de Desenvolvimento (BID), junto à Organização para Cooperação e Desenvolvimento Econômico (OCDE) e Banco Mundial (BM).

**Resultados:**

O número total de mortes por DTNs na AS, no ano de 2019, foi de 13.635, 5.450 óbitos nas faixas etárias produtivas de acordo, 40% do total de mortes relacionadas às DTNs nesse continente. Quanto aos (YPLL), perderam-se 93.657 anos, afetando mais os homens (58.815 anos) do que as mulheres (34.842 anos). A perda permanente de produtividade total foi de aproximadamente US$ 132 milhões e US$ 281 milhões em perda de produtividade permanente (PPP). O custo por morte foi de US$ 28.087. A variação nos cenários aponta para robustez nas estimativas, mesmo com diferenças significativas entre os países.

**Conclusão:**

A baixa mortalidade das DTNs, doenças que se tornam crônicas e debilitam, aumenta em longo prazo os encargos sociais e econômicos sobre indivíduos, famílias e governos. Conclui-se que, por imporem menor encargo econômico e social à maioria dos países da AS do que outros grupos de doenças, as DTNs tornam-se ainda mais negligenciadas. Apesar do esforço global promovido pela Organização Mundial da Saúde (OMS) para fomentar intervenções nas DTNs, são necessários mais estudos econômicos, a fim de avaliar o impacto das intervenções nessas doenças.

